# Gene editing tools for mycoplasmas: references and future directions for efficient genome manipulation

**DOI:** 10.3389/fmicb.2023.1191812

**Published:** 2023-05-18

**Authors:** Gang Zhao, Doukun Lu, Min Li, Yujiong Wang

**Affiliations:** ^1^Key Laboratory of Ministry of Education for Conservation and Utilization of Special Biological Resources in the Western China, Yinchuan, China; ^2^School of Life Sciences, Ningxia University, Yinchuan, China; ^3^National Key Laboratory of Agricultural Microbiology, Huazhong Agricultural University, Wuhan, China

**Keywords:** mycoplasma, genome engineering, transposon, clustered regularly interspaced short palindromic repeats/Cas9 system, synthetic biology

## Abstract

Mycoplasmas are successful pathogens that cause debilitating diseases in humans and various animal hosts. Despite the exceptionally streamlined genomes, mycoplasmas have evolved specific mechanisms to access essential nutrients from host cells. The paucity of genetic tools to manipulate mycoplasma genomes has impeded studies of the virulence factors of pathogenic species and mechanisms to access nutrients. This review summarizes several strategies for editing of mycoplasma genomes, including homologous recombination, transposons, clustered regularly interspaced short palindromic repeats (CRISPR)/Cas system, and synthetic biology. In addition, the mechanisms and features of different tools are discussed to provide references and future directions for efficient manipulation of mycoplasma genomes.

## 1. Introduction

Mollicutes (“mycoplasmas”) are host-restricted prokaryotes and the simplest self-replicating organisms evolved from Gram-positive ancestors, which are characterized by a low GC content, small genomes (0.6–1.35 Mb), no cell wall, reduced coding capacity, and limited metabolic capacities (Sirand-Pugnet et al., 2007). Despite undergoing reductive evolution, mycoplasmas primarily colonize the mucosa of the respiratory and urogenital tracts, as well as the joints of vertebrate hosts, including multiple livestock, wild animal species, and humans. Some mycoplasmas are successful pathogens capable of establishing infection, which can result in significant socioeconomic consequences (Razin et al., 1998; Rosengarten et al., 2001; Citti and Blanchard, 2013; Arfi et al., 2021). Intrinsic antibiotic resistance, rapid tolerance to chemotherapeutic agents, and co-infection with other pathogenic species are causing growing concerns of mycoplasmas in both the medical and veterinary fields (Gautier-Bouchardon, 2018; Chen et al., 2023).

The growing body of sequence data has improved understanding of the structure and dynamics of mycoplasmas. However, genomic studies have revealed that mycoplasmas lack the classical repertoire of virulence genes common to pathogenic species. The molecular mechanisms underlying the pathogenesis of mycoplasmas in host calls include adhesion to the host respiratory epithelium, cell damage caused by cytotoxic metabolic compounds and the releasome, and modulation of the host microbicidal response. Experimentally confirmed virulence factors of mycoplasmas include the community-acquired respiratory distress syndrome toxin, hydrogen peroxide, and hydrogen sulfide (Burki et al., 2015; Benedetti et al., 2020; Leal Zimmer et al., 2020; Gaurivaud and Tardy, 2022). Limited information about the classical repertoire of virulence genes has impeded identification of the virulence genes and elucidation of the pathogenesis of mycoplasmas. In addition, the lack of appropriate genetic tools for genome manipulation has limited research on the virulence factors of mycoplasmas. Transposon-based vectors have been successfully applied for random insertion and inactivation of target genes of the mycoplasma genome (Dybvig et al., 2000; Kenri et al., 2004; Baranowski et al., 2010; Rideau et al., 2019). Over the past decade, synthetic biology has been successfully applied to several phylogenetically related species, but yet limited with other species (Gibson et al., 2008a; Schieck et al., 2016). Recently, the clustered regularly interspaced short palindromic repeats (CRISPR)/CRISPR-associated protein 9 (Cas9) system has been successfully applied to silence target genes in the mycoplasma genome (Mariscal et al., 2018; Evsyutina et al., 2022). However, a more accurate, stable, and efficient genetic editing tool is urgently needed.

Natural plasmids of mycoplasmas are transmitted between species sharing a common host (Breton et al., 2012). *OriC* plasmids were developed to examine gene function in mycoplasmas, mesoplasmas, and spiroplasmas (Halbedel and Stulke, 2007; Maglennon et al., 2013; Renaudin et al., 2015; Matteau et al., 2017). In addition to these plasmids, this review summarizes current genetic tools to manipulate the mycoplasma genome. Current applications are classified as homologous recombination (HR), transposons, CRISPR/Cas systems, and synthetic biology. In addition, the development and optimization of CRISPR/Cas systems as novel genome editing tools for mycoplasmas are discussed.

## 2. HR

HR is essential to access redundant genetic information encoded by sister chromatids or homologous chromosomes when both strands of the DNA double helix are compromised to support DNA replication and repair double-strand breaks (DSBs) (Wright et al., 2018). The mechanism of HR to repair DSBs has been widely applied for editing of bacterial genomes, but relatively few studies have investigated HR in mycoplasmas. Nonetheless, HR has been successfully used to edit the genomes of various *Mycoplasma* species, including *M. gallisepticum* (Cao et al., 1994; Lee et al., 2008), *M. genitalium* (Dhandayuthapani et al., 1999, 2001), *M. capricolum* subsp. *capricolum* (*M. capricolum*) (Janis et al., 2005), *M. pneumoniae* (Krishnakumar et al., 2010), and *M. hyopneumoniae* (Clampitt et al., 2021). HR was also used to integrate homologous DNA through a plasmid into the mycoplasma genome. However, success of this process was very low because of the lack of an efficient recombinase and transformation procedures. Therefore, an exogenous recombinase was applied to edit the genome of *Mycoplasma gallisepticum* to overcome the low efficiency of HR. The recE and recT genes of *Bacillus subtilis* were cloned into transposon-based vectors and integrated into the genome after transformation. The recombination templates in *oriC* plasmids were transformed into *M. gallisepticum* strains expressing RecE and RecT to generate a RecET-like system for precise recombination events leading to short deletions, the addition of resistance genes, and replacement of short genome regions (Ipoutcha et al., 2022). An oligonucleotide “recombineering” method for *M. pneumoniae* was also developed with the GP35 recombinase of *B. subtilis* to generate point mutations or deletion of larger fragments. Then, the CRISPR/Cas9 system was used to counter-select non-edited cells (Pinero-Lambea et al., 2020). In addition, the recombinase RecA of *Escherichia coli* was shown to enhance targeted HR in *Mycoplasma mycoides* subsp. *capri* and *M. hyorhinis* (Allam et al., 2010; Ishag et al., 2017).

## 3. Transposons

Transposons are mobile genetic elements that evolved to execute highly efficient integration of genes into the host genome. As the most prominent mechanism, a transposase mediates excision of an element from the donor location and facilitates integration into a different locus of the genome (Sandoval-Villegas et al., 2021). Transposons are also widely used to integrate genes into the host genome. The transposons Tn*916* and Tn*4001*, in addition to related derivatives, have been successfully used in Mollicutes (Halbedel and Stulke, 2007). Transposon Tn*916*, which was originally isolated from *Enterococcus faecalis*, is a conjugative 18-kb transposable element containing the *xis-Tn*/*int-Tn* genes for excision/integration, the *tetM* tetracycline resistance genes, a set of genes for intercellular transfer, and two imperfect inverted repeat sequence (20–60 bp) at both ends (Franke and Clewell, 1981; Clewell et al., 1988, 1995). Transposon Tn*4001*, originally isolated from *Staphylococcus aureus*, is a 4.5-kb composite containing an IS256 sequence at both ends of the *aac-aphD* gene, which confers resistance to gentamicin, kanamycin, and tobramycin (Lyon et al., 1984).

Integration of a transposon into the mycoplasma and acholeplasma genomes was first reported in *Mycoplasma pulmonis* and *Acholeplasma laidlawii*, respectively (Dybvig and Cassell, 1987). However, integration of Tn*916* occurs at preferred hot spots and, thus, is less suitable for saturating transposon mutagenesis (Nelson et al., 1997). In 1989, Tn*4001* was first integrated into the *M. pulmonis* genome, although the transposase was deleted to prevent reintegration and loss of the transposon by re-excision. The derivative Tn*4001* is also called mini-Tn*4001* (Zimmerman and Herrmann, 2005). To date, transposons have been widely used to construct mutant libraries of several mycoplasmas. For instance, Tn*4001* and mini-Tn*4001* were used to construct mutant libraries of *M. pneumoniae* to screen for essential genes and those associated with gliding motility (Hasselbring et al., 2006; Lluch-Senar et al., 2015). Tn*916* and Tn*4001* were also used to construct two mutant libraries of *M. gallisepticum* to screen for genes that regulate biofilm formation (Whetzel et al., 2003; Wang et al., 2017). In addition, mini-Tn*4001* was applied to construct mutant libraries of *M. agalactiae* and *M. bovis* to identify genes that regulate nutrient acquisition from cells, genes that affect colonization and diffusion in the host cell, as well as other essential genes, such as those that code for adhesin proteins (Baranowski et al., 2010, 2014; Hegde et al., 2016; Josi et al., 2019; Zhu et al., 2020). Furthermore, mini-Tn*4001*, Tn*4001*, and Tn*916* were successfully used to construct mutant libraries to identify the essential genes of various species of mycoplasmas, including *M. genitalium*, *M. bovis*, *M. pulmonis*, *M. hyopneumoniae*, *M. mycoides*, *M. hominis*, and *M. hyorhinis*, in addition to *Ureaplasma parvum* (Hutchison et al., 1999; Glass et al., 2006; French et al., 2008; Maglennon et al., 2013; Aboklaish et al., 2014; Sharma et al., 2014; Hutchison et al., 2016; Rideau et al., 2019; Trueeb et al., 2019). Also, mini-Tn*4001* was used to express mCherry, Mko2, and mNeonGreen in *M. bovis* and *M. mycoides* subsp. *mycoides* to investigate host-pathogen interactions (Bonnefois et al., 2016). Tn*4001* was also applied to examine the localization of green fluorescent protein (GFP)-tagged proteins of *M. pneumoniae* in mycoplasma cells (Kenri et al., 2004).

The transformation efficiency of Tn*4001* is reported to significantly vary among different species, resulting in differences in transposition efficiency. The EF-Tu regulatory region of mycoplasma species was cloned into a transposon-based vector to regulate transposase expression and the antibiotic resistance marker to overcome this disadvantage. This derivative transposon, named SynMyco, was shown to increase transformation efficiency in *M. gallisepticum*, *M. feriruminatoris*, and *M. agalactiae*, but not *M. pneumoniae* (Montero-Blay et al., 2019). Recently, a LoxTnSeq system was developed to delete a large random genome fragment. Sequences of *loxP* were inserted into the genome of *M. pneumoniae* combined with expression of the exogenous recombinase Cre, while the large DNA fragment with a *loxP* sequence at both ends was deleted (Shaw et al., 2020). In addition, Tn*5* was applied to generate mutant libraries of *M. mycoides*, *Spiroplasma citri*, and *Mesoplasma florum* (Mutaqin et al., 2011; Hutchison et al., 2016; Baby et al., 2018).

## 4. CRISPR/Cas system

CRISPR-based genetic tools have revolutionized the field of genome engineering in eukaryotes and prokaryotes since first introduced in 2012. The classical CRISPR/Cas9 system uses a single-guide RNA (sgRNA) to target Cas9 nuclease to the desired DNA locus and create a site-specific DSB to the DNA. Genome editing is dependent on the repair machinery of the cell, including non-homologous end joining and homology-directed repair of DSBs (Jinek et al., 2012). Both endogenous and exogenous CRISPR/Cas systems have been applied to edit mycoplasma genomes (Table 1). In addition, the exogenous CRISPR/Cas system has been used to kill mycoplasma. Due to the lack of efficient non-homologous end joining and homology-directed repair pathways to repair DSBs, the CRISPR/Cas9 system was applied to break the genome of *M. pneumoniae* and limit growth (Broto et al., 2022). This system was also used to counter-select non-edited mycoplasma cells and to recover edited mycoplasma clones with limited screening of surviving cells (Pinero-Lambea et al., 2020). Moreover, an endogenous CRISPR/Cas system was used to edit the *ksgA* and *munA* genes of *M. gallisepticum*. The endogenous Cas protein of *M. gallisepticum*, which is reported to cut DNA, shares 26% amino acid sequence similarity with the type II-A Cas9 of *S. aureus* (Mahdizadeh et al., 2020; Klose et al., 2022). Bioinformatics revealed that the genome carried a gene with significant homology with the *Ku* and *LigD* genes of *B. subtilis*, which are the key elements of the non-homologous end joining repair system. These findings demonstrate that DNA repair systems differ among mycoplasma species. Further studies of DNA repair systems will contribute to further applications of the CRISPR/Cas system for genome editing of mycoplasmas.

**Table 1:** Applications of CRISPR/Cas system in mycoplasma.

Name	Cas9 Source	Cas9 Activity	Mechanism	Application	Species (reference)
CRISPR/Cas9	Exogenous	Active	DSB toxicity	Counter select	*M. pneumoniae* (Pinero-Lambea et al., 2020; Broto et al., 2022)
CRISPR/Cas9	Endogenous	Active	NHEJ (putative)	Knock-out	*M. gallisepticum* (Mahdizadeh et al., 2020; Klose et al., 2022)
Cas9-Base Editor	Exogenous	Inactive	Base edit	Knock-out	*Mmm*^a^ (Lpoutcha et al., 2022) *M. bovis* (Lpoutcha et al., 2022) *M. gallisepticum* (Lpoutcha et al., 2022)
CRISPRi	Exogenous	Inactive	Interfere	Knock-down	*M. hominis* (Evsyutina et al., 2022) *M. gallisepticum* (Evsyutina et al., 2022)
Inducible CRISPRi	Exogenous	Inactive	Interfere	Knock-down	*M. pneumoniae* (Mariscal et al., 2018) *M. mycoides* (Mariscal et al., 2018)

^a^*M. mycoides* subsp. mycoides.

Considering the low efficiency of repairing DSBs, endonuclease-deficient Cas9 (dCas9)-based gene editing tools, including CRISPR interference (CRISPRi), DNA base editing, and inducible CRISPRi, were applied to edit the genomes of mycoplasmas. Cas9 base-editing systems have been constructed to multiply the genome without inducing DSBs. DNA base editing systems combine a catalytically inactive form of Cas9 fused with a cytosine deaminase (CBE). This Cas9/CBE fusion protein is guided to specific loci by sgRNAs, where CBE catalyzes deamination of cytosine into uracil, which is recognized as thymine after replication. The C:G to A:T transition allows for insertion of a stop codon into the genome of *M. bovis*, *M. mycoides* subsp. *mycoides*, and *M. gallisepticum*. In addition, whole-genome sequencing revealed that this genome editing system is efficient with limited induction of off-target mutations (Lpoutcha et al., 2022). For the CRISPRi system, the dCas9 is guided to the loci by sgRNA and inhibits expression of the target gene by interfering with transcriptional elongation by binding of RNA polymerase or associated transcription factors (Qi et al., 2013). A single-plasmid transposon-based CRISPRi system was applied for genome editing of *M. gallisepticum* and *M. hominis* (Evsyutina et al., 2022). In this paper, the inactive Cas9 of *Streptococcus pyogenes* was cloned into the transposon-based vector pRLM5L2 and expressed in mycoplasma cells. However, expression of dCas9 had no significant effect on the growth rate of mycoplasma cells (Evsyutina et al., 2022). Moreover, an inducible CRISPRi system was also developed for *M. pneumoniae* and *M. mycoides*. With this system, the tetracycline operator regulates expression of the exogenous inactive form of Cas9, whereas tetracycline is used to induce expression of dCas9 and inhibit expression of the target gene (Mariscal et al., 2018). However, the effect of exogenous Cas9/dCas9 on the growth of mycoplasma when used to edit the genome remains unclear.

## 5. Synthetic biology strategies

Synthetic biology strategies, which use yeast cells to engineer and transfer a bacterial genome into a recipient cell, have been applied to edit the genomes of *M. genitalium*, *M. mycoides* subsp. *capri*, and *M. mycoides* (Table 2), although gene editing is dependent on genome synthesis (Gibson et al., 2008a,b; Lartigue et al., 2009; Gibson et al., 2010). As compared to a synthetic genome, the genomes of *M. genitalium*, *M. pneumoniae*, *M. mycoides* subsp. *capri*, *M. hominis*, *Mesoplasma florum*, and *A. laidlawii* were cloned into yeast cells as circular centromeric plasmids (Karas et al., 2012; Rideau et al., 2017; Baby et al., 2018). Then, the restriction endonuclease *Asc*I was used to create DSBs in the circular plasmids and a DNA fragment was inserted using HR in yeast cells. The mycoplasma genome appeared stable in yeast cells and provided a platform to engineer the mycoplasma genome *in vivo* (Benders et al., 2010). Besides cloning of the mycoplasma genome in yeast cells, the whole genome integrated with a yeast vector was transformed (Lartigue et al., 2009). Based on this platform, TREC (tandem repeat coupled with endonuclease cleavage), RMCE (Cre/*loxP*-based recombinase-mediated cassette exchange), TREC-IN (TREC-assisted gene knock-in), CreasPy-Cloning, Marker-less/driven, and meiotic recombination methods were developed to edit the genome of mycoplasmas in yeast cells. The TREC method uses a DNA cassette containing a knock-out CORE consisting of an 18-bp I-*Sce*I recognition site, the *SCEI* gene under the control of the *GAL1* promoter, and the *URA3* marker. A DNA fragment homologous to the sequence upstream of the target site was used to insert into the genome of *M. genitalium* by HR in yeast cells, which generated tandem repeat sequences flanking the knock-out CORE. The inducible expression of I-*Sce*I generated DSBs and promoted intra-molecular HR between the repeat sequences for excision of the CORE (Noskov et al., 2010; Schieck et al., 2016). The RMCE method uses a DNA cassette containing the *cre* gene under the control of the *GAL1* promoter and *URA3* marker flanked by the *loxP* sequence, where inducible expression of the Cre recombinase was used to insert DNA fragments into the mycoplasma genome (Noskov et al., 2010, 2015). The TREC-IN method was developed based on the TREC method and used to insert the target gene into the mycoplasma genome, where the gene was located at the 3′ end of the knock-out CORE and remained in the genome after excision of the CORE (Chandran et al., 2014; Lartigue et al., 2019). The CreasPy-Cloning method is a recently developed approach for simultaneous cloning and engineering of mycoplasma genomes in yeast cells. This method combines the abilities of Cas9 to cleave DNA at a specific locus and the efficient HR of yeast cells to edit the mycoplasma genome, which was transformed into yeast cells in whole (Kannan et al., 2016; Tsarmpopoulos et al., 2016; Rideau et al., 2017; Ruiz et al., 2019; Talenton et al., 2022). The Marker-less/driven method with EZ-Tn5™ transposase was used to successfully insert DNA fragments into the genome of *M. mycoides in vitro* and then the edited genome was transformed into the mycoplasma (Karas et al., 2014). The meiotic recombination method replaces the individual gene with the GFP marker in the mycoplasma genome by HR in yeast cells. Then, the yeast cells with GFP markers at different loci are mixed and the progressively clustering genomic segments are deleted by meiotic recombination between the mycoplasma genomes harbored in yeast cells (Sugiyama et al., 2005; Suzuki et al., 2011, 2015).

**Table 2:** Application of synthetic biology in mycoplasma.

Name	Strategy and type of modifications	Species (reference)
Synthetic approach	Synthetic genome, deletion and insertion	*M. genitalium* (Gibson et al., 2008a,b) *Mmc*^a^ (Gibson et al., 2010)
*Asc*I	HDR repairs the DSB created by restriction endonuclease *Asc*I, insertion	*M. genitalium* (Rideau et al., 2017) *Mmc*^a^ (Rideau et al., 2017) *M. pneumoniae* (Rideau et al., 2017)
TREC	HDR repairs the DSB created by I-*Sce*I, deletion	*M. genitalium* (Benders et al., 2010) *Mmc*^a^ (Schieck et al., 2016)
RMCE	Cre recombinase insert DNA fragment into the genome, insertion	*M. genitalium* (Benders et al., 2010) *M. mycoides* (Noskov et al., 2010)
TREC-IN	HDR repair the DSB created by I-*Sce*I, insertion	*Mmc*^a^ (Chandran et al., 2014; Noskov et al., 2015)
CreasPy-Cloning	HDR repair the DSB created by Cas9, deletion	*M. hominis* (Baby et al., 2018) *M. pneumoniae* (Lartigue et al., 2019) *M. feriruminatoris* (Ruiz et al., 2019) *Mmc^a^* (Talenton et al., 2022) *M. mycoides* (Tsarmpopoulos et al., 2016)
Marker-less/driven	Tn*5* transposase edit gene *in vitro*, deletion	*M. mycoides* (Kannan et al., 2016)
Meiotic recombination	Delete genomic segments by using meiotic recombination, deletion	*M. mycoides* (Suzuki et al., 2011; Karas et al., 2014; Suzuki et al., 2015)
RAGE	Cre recombinase insert DNA fragment into the genome, insertion	*M. pneumoniae* (Sugiyama et al., 2005)

^a^*M. mycoides* subsp. capri.

Each of the above methods requires transfer of the edited genome into the recipient cell to generate mutants. Such transfer is possible for several species related to the *M. mycoides* cluster, but not members of other phylogenetic groups. The recombinase-assisted genomic engineering (RAGE) method was developed to overcome this disadvantage with genome editing of *M. pneumoniae*. This method uses the transformation-associated recombination mechanism, where a DNA fragment of the mycoplasma genome, the *cre* gene, the selection marker *aac-aph*, and a linearized bacterial artificial chromosome-yeast artificial chromosome shuttle vector are assembled in a recombinant plasmid in yeast cells. Then, the plasmid is extracted and transformed into *E. coli* cells for amplification. Thereafter, *M. pneumoniae* cells are transformed with numerous plasmids and the genome is edited by RMCE. Based on this new strategy, the TREC, Cre/*loxP*, TREC-IN, and CreasPy-Cloning methods could be used to edit fragments of mycoplasma genomes in yeast cells (Garcia-Morales et al., 2020).

Lastly, the “targeting-induced local lesions in genomes” method is a reverse-genetic method to edit the *M. hominis* genome, which combines point mutations (C-G to T-A) induced by ethyl methane sulfonate with a DNA screening technique to generate a library of *M. hominis* mutants (Pereyre et al., 2018).

## 6. Current thoughts of improvement of existing tools

**Figure 1:**
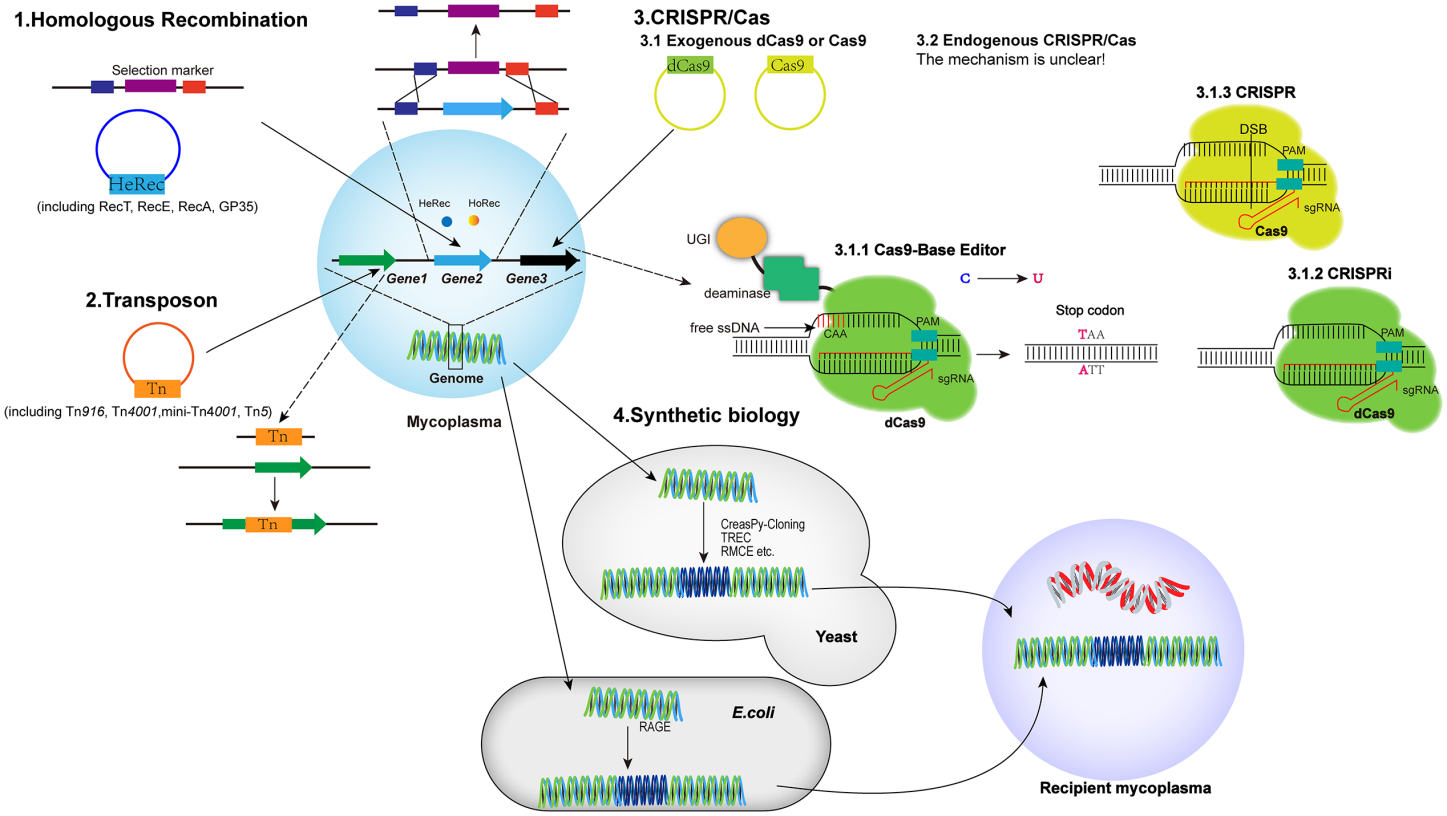
Schematics of the genetic tools based on HR, transposons, the CRISPR/Cas9 system, and synthetic biology. HR: the heterologous recombinase (HeRec) and homologous recombinase (HoRec) were applied to edit the mycoplasma genome. Transposon: Tn*4001*, mini-Tn*4001*, Tn*5*, and Tn*916* were used to insert transposons into the genome for gene knock-out. CRISPR/Cas9: the endogenous CRISPR/Cas system of *M. gallisepticum* and exogenous CRISPR/Cas9 system were used to edit the mycoplasma genome. The exogenous CRISPR/Cas9 system includes: (i) inactivated Cas9 fused with a CBE to induce C:G to T:A for insertion of a stop codon into the target gene; (ii) inactivated Cas9 for interfering with expression of the target gene; and (iii) activated Cas9 for knock-out of the target gene. Synthetic biology: the genome was edited by the TREC, CreasPy-Cloning, and RMEC methods in yeast cells and then transferred into recipient mycoplasma cells; the genome fragments of *M. pneumoniae* were edited by RAGE in *E. coli* cells and then transferred into *M. pneumoniae* recipient cells.

Due to the lack of efficient genetic tools, it is challenging to elucidate the functional genomics of mycoplasmas. Although several methods have been developed to knock-out or knock-down target genes in the genome (Figure 1; Table 3), stable, efficient, and universal genetic tools have not yet been established for mycoplasmas.

**Table 3:** The list of validated method for genetic editing in mycoplasma.

Species	Methods			
	Homologous recombination	Transposon	CRISPR/Cas	Synthetic biology
*M. pneumoniae*	HR (Krishnakumar et al., 2010), GP35 (Pinero-Lambea et al., 2020), Cre (Sugiyama et al., 2005)	Tn*4001* (Hasselbring et al., 2006), mini-Tn*4001* (Lluch-Senar et al., 2015)	CRISPR/Cas9 (Pinero-Lambea et al., 2020; Broto et al., 2022), Inducible CRISPRi (Mariscal et al., 2018)	*Asc*I (Rideau et al., 2017), CreasPy-Cloning (Lartigue et al., 2019), RAGE (Sugiyama et al., 2005)
*M. hominis*		mini-Tn*4001* (Rideau et al., 2019)	CRISPRi (Evsyutina et al., 2022)	CreasPy-Cloning (Baby et al., 2018)
*M. genitalium*	HR (Dhandayuthapani et al., 1999, 2001)	Tn*4001* (Hutchison et al., 1999)		Synthetic approach (Gibson et al., 2008a,b), *Asc*I (Rideau et al., 2017), TREC (Benders et al., 2010), RMCE (Benders et al., 2010)
*Ureaplasma parvum*		mini-Tn*4001* (Aboklaish et al., 2014)		
*Mmm* ^a^			Cas9-Base Editor (Lpoutcha et al., 2022)	
*Mmc* ^b^	RecA (Allam et al., 2010)			Synthetic approach (Gibson et al., 2010), *AscI* (Rideau et al., 2017), TREC (Schieck et al., 2016), TREC-IN (Chandran et al., 2014; Noskov et al., 2015)
*M. capricolum* subsp. *capricolum*	HR (Janis et al., 2005)			
*M. bovis*		mini-Tn4*001* (Josi et al., 2019; Zhu et al., 2020) Tn*4001* (Sharma et al., 2014)	Cas9-Base Editor (Lpoutcha et al., 2022)	
*M. agalactiae*		mini-Tn*4001* (Baranowski et al., 2010, 2014; Hegde et al., 2016), SynMyco transposon (Montero-Blay et al., 2019)		
*M. mycoides* ^c^		Tn*4001* (Hutchison et al., 2016), Tn*5* (Hutchison et al., 2016; Kannan et al., 2016)	Inducible CRISPRi (Mariscal et al., 2018)	RMCE (Noskov et al., 2010), Marker-less/driven (Kannan et al., 2016), Meiotic recombination (Suzuki et al., 2011; Karas et al., 2014; Suzuki et al., 2015), CReasPy-cloning (Talenton et al., 2022)
*M. feriruminatoris*		SynMyco transposon (Montero-Blay et al., 2019)		CReasPy-cloning (Ruiz et al., 2019)
*M. gallisepticum*	HR (Cao et al., 1994; Lee et al., 2008), RecET-like system (Ipoutcha et al., 2022)	Tn*916* (Whetzel et al., 2003), mini-Tn*4001* (Wang et al., 2017), SynMyco transposon (Montero-Blay et al., 2019)	CRISPR-Cas9 (Mahdizadeh et al., 2020; Klose et al., 2022), Cas9-Base Editor (Lpoutcha et al., 2022), CRISPRi (Evsyutina et al., 2022)	
*M. hyorhinis*	RecA (Ishag et al., 2017)	mini-Tn*4001* (Trueeb et al., 2019)		
*M. hyopneumoniae*	HR (Clampitt et al., 2021)	mini-Tn*4001* (Maglennon et al., 2013; Trueeb et al., 2019)		
*M. pulmonis*		Tn*916* (Dybvig and Cassell, 1987), Tn*4001* (French et al., 2008)		
*Acholeplasma laidlawii*		Tn*916* (Dybvig and Cassell, 1987)		
*Mesoplasma florum*		Tn*5* (Mutaqin et al., 2011)		
*S. citri*		EZ-Tn*5*™ (Baby et al., 2018)		

^a^*M. mycoides* subsp. mycoides. ^b^*M. mycoides* subsp. capri. ^c^*M. mycoides* is an artificial mycoplasma based on *M. mycoides* subsp. capri. HR, Homologous recombination; TREC, Tandem repeat coupled with endonuclease cleavage; TREC-IN, TREC-assisted gene knock-in; RMCE, Cre/loxP-based Recombinase-Mediated Cassette Exchange; RAGE, Recombinase-assisted genomic engineering; EZ-Tn5™, Transposon tools based on a hyperactive Tn5 transposition system.

### 6.1. Development of efficient genetic tools for knock-out of target genes in mycoplasmas

**Figure 2:**
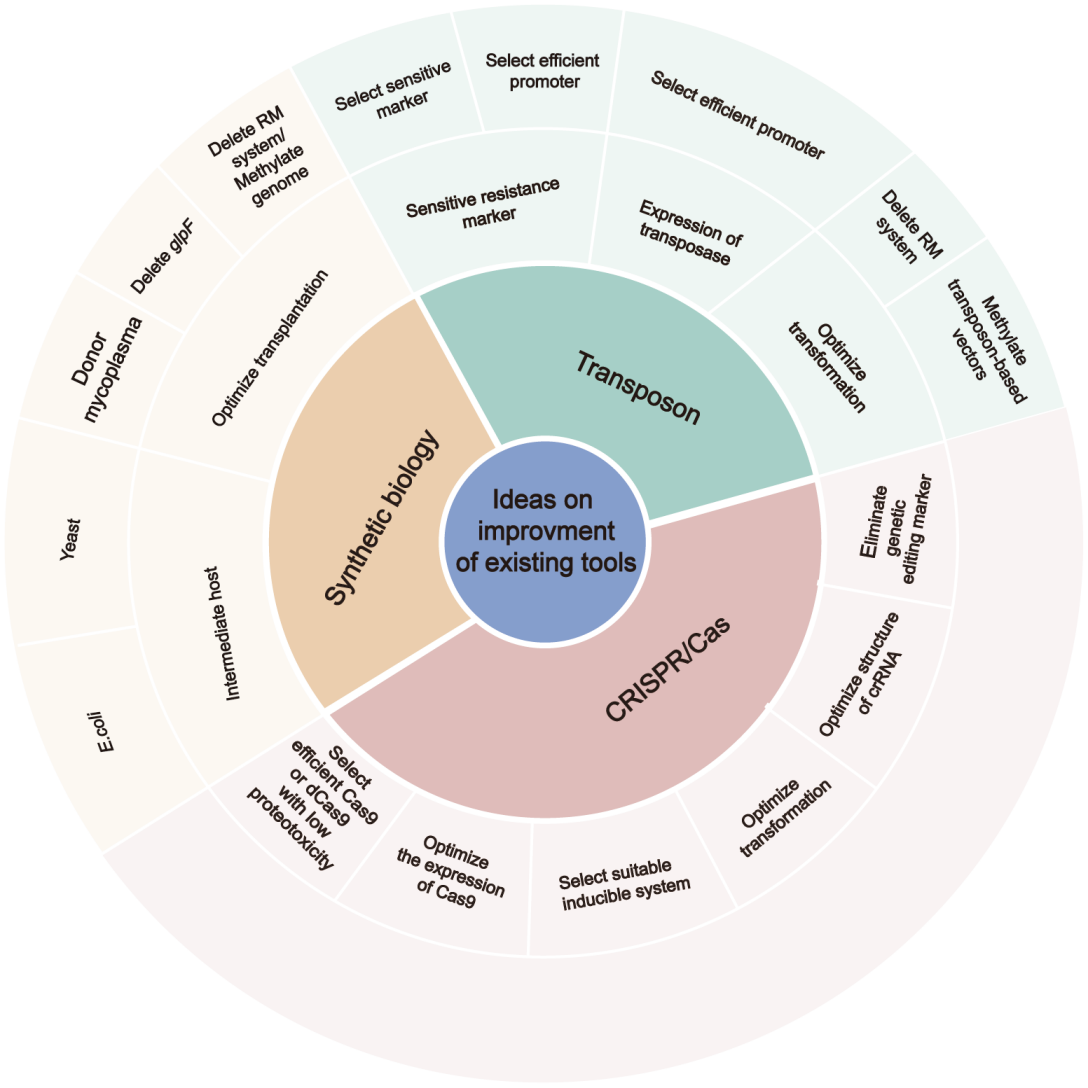
The ideas on improving the efficient of existing tools for editing of mycoplasma genomes. RM, restriction-modification; crRNA, CRISPR RNA.

Transposons are widely used to generate mutant libraries of mycoplasmas and combined with high-throughput sequencing to identify the inserted loci. However, transposon mutagenesis is inefficient for some mycoplasma species due to integration site preferences, effects on the expression of neighboring genes through homology-based silencing or read-through activity of regulatory elements, and truncation of the target gene (Rebollo et al., 2012). In addition, the resistance marker of the transposon, the restriction-modification system of the mycoplasma, and the expression of transposase and resistance marker could influence the success of transposon mutagenesis (Dybvig et al., 2000; Calcutt and Foecking, 2015; Montero-Blay et al., 2019; Rideau et al., 2019). Therefore, sensitive antibiotic markers and suitable promoters should be selected to induce expression of the transposon and antibiotic marker, and methylate the transposon-based vectors by methyltransferase when transposons are used to generate a mutant library of a new mycoplasma species (Figure 2).

A synthetic biology strategy was developed for site-directed mutagenesis in mycoplasmas. This method allows efficient editing of the mycoplasma genome in yeast cells. Several studies revealed that deletion of the glycerol uptake facilitator protein gene (*glpF*) and direct cell-to-cell transfer of the mycoplasma genome to a yeast cell could promote the genome transfer process (Karas et al., 2014, 2019). However, genome transplantation limits the popularization and application of this method, which has only been successful with *M. capricolum* subsp. *capricolum* as the recipient cell (Labroussaa et al., 2016). Transfer of the *M. florum* genome indicates that this method has potential for other organisms besides mycoplasma species related to the *M. mycoides* cluster. Nonetheless, some strategies have been developed to increase the efficiency of genome transfer, which include genome methylation, deletion of restriction systems, and using mycoplasma as donor for transformation (Karas et al., 2013, 2014, 2019). Therefore, the RAGE method uses *M. pneumoniae* as recipient cells (Garcia-Morales et al., 2020).

Besides the synthetic biology strategy, the CRISPR/Cas system was developed to induce site-directed mutagenesis and was recently applied in various mollicutes, including the main pathogens of humans, ruminants, and plants (Ipoutcha et al., 2019). However, successful editing of the mycoplasma genome was limited to the endogenous CRISPR/Cas system of *M. gallisepticum* (Klose et al., 2022). Notably, the unclear characterization of the endogenous CRISPR/Cas9 systems and the requirement of a protospacer adjacent motif resulted in unpredictable results and impeded further modification of the system. Several studies have reported that the exogenous Cas9 of *S. pyogenes* was able to generate DSBs in the mycoplasma genome. Due to the lack of an efficient system to repair DSBs, the exogenous CRISPR/Cas9 system was used to inhibit mycoplasma growth or counter-select non-edited mycoplasmas to recover edited mycoplasma clones with limited screening of surviving cells.

Bioinformatics revealed that some mycoplasma genomes carry homologs of genes responsible for DNA repair in other bacteria, including those for the SOS stress response, recombinational repair, base excision repair, and nucleotide excision repair. However, there is limited experimental evidence of these DNA repair-associated genes in mycoplasmas (Carvalho et al., 2005; Burgos et al., 2012). As the exogenous recE/recT recombinase and GP35 recombinase of *B. subtilis* were found to function in mycoplasmas, future studies are warranted to investigate the combination of the exogenous CRISPR/Cas system and an exogenous recombinase to edit genes of mycoplasmas. The efficient dCas9-Base editor system was recently applied to induce mutations without generating DSBs. Although the exogenous CRISPR/Cas9 system can knock-out genes, various factors should be considered, including (i) the effect of exogenous Cas9/dCas9 proteotoxicity on mycoplasma growth; (ii) regulation of gene expression by exogenous Cas9/dCas9; (iii) identification of a suitable inducible promoter for Cas9/dCas9; and (iv) the development of a marker-less genetic editing system (Figure 2). Five inducible systems have been successfully applied in mycoplasmas, which include the riboswitch, the TetR transcription regulator of *B. subtilis*, the lac operon of *E. coli*, the CI protein of bacteriophage lambda, and the AraR transcription regulator of *B. subtilis* (Mariscal et al., 2018; Broto et al., 2022).

### 6.2. Development of efficient genetic tools for knock-down of target genes in mycoplasmas

The evolution of mycoplasmas involved a degenerative process where the genomes have high proportions of nonredundant genes essential for cell growth and proliferation. About 81, 64, and 52% of the genes of *M. genitalium*, *M. pneumoniae*, and *M. mycoides*, respectively, are considered essential (Glass et al., 2006, 2017), as compared to only 7% of the genes of *E. coli*, as determined in a targeted knock-out study (Baba et al., 2006). These data demonstrate that a large number of genes are not suitable for knock-out. The CRISPRi and inducible CRISPRi systems were recently developed for knock-down of target genes. Due to the complicated transcriptome of mycoplasmas (e.g., 40.7% of operons are polycistronic) (Guell et al., 2009), the major disadvantage of the CRISPRi system is the polar effects on genes upstream and downstream from the target in an operon (Peters et al., 2016). The diverse orthologues of Cas9 exhibit different knock-down efficiencies and proteotoxicities in mycobacteria (Rock et al., 2017). Atypical CRISPR RNA (crRNA) are more efficient than typical crRNA (Petassi et al., 2020) and continuous expression of Cas9 by integration into the genome increased the knock-down efficiency (Peters et al., 2019). In consideration of the effect of these factors on the efficiency of the CRISPRi system in other organisms, an efficient CRISPRi system for mycoplasma could be achieved by selecting highly efficient Cas9 with low proteotoxicity and a suitable inducible promoter, while optimizing the structure of the crRNA, as well as the induction conditions, and construction of a stable plasmid carrying Cas9 or integrating Cas9 into the genome (Figure 2).

## 7. Conclusion

Transposons are conventionally used to generate mutant libraries, which can be combined with smart screening systems to identify genes that regulate nutrient acquisition from host cells, affect colonization and diffusion in the host cell, as well as other essential genes, such as those that code for adhesin proteins. However, the lack of genetic tools for site-directed mutagenesis in many mycoplasma species has impeded further clarification of the interactions between mycoplasmas and host cells. The CRISPR/Cas system and synthetic biology were recently applied to knock-out target genes. These studies provide new insights into the genetic manipulation of mycoplasmas. Notably, the CRISPRi system is more appropriate than knock-down/out methods to analyze the functions of essential genes. In addition, gene function can be confirmed by trans-complementation of mutants based on *oriC* plasmids. This review summarizes the results of recent studies and discusses strategies for the development of accurate and efficient tools for editing of mycoplasma genomes.

## Author contributions

GZ and DL write original manuscript. ML and YW designed and revised the article. All authors contributed to the article and approved the submitted version.

## Funding

This work was supported by the Youth Program of National Natural Science Foundation of China (#32102672), National Natural Science Foundation of China Joint Fund Project (U22A20505).

## Conflict of interest

The authors declare that the research was conducted in the absence of any commercial or financial relationships that could be construed as a potential conflict of interest.

## Publisher’s note

All claims expressed in this article are solely those of the authors and do not necessarily represent those of their affiliated organizations, or those of the publisher, the editors and the reviewers. Any product that may be evaluated in this article, or claim that may be made by its manufacturer, is not guaranteed or endorsed by the publisher.
